# Time series analysis comparing mandatory and voluntary notification of newly diagnosed HIV infections in a city with a concentrated epidemic

**DOI:** 10.1186/1471-2458-13-338

**Published:** 2013-04-12

**Authors:** Juliana M Reyes-Urueña, Patricia García de Olalla, Santiago Perez-Hoyos, Joan A Caylà

**Affiliations:** 1 Epidemiology Service, Agencia de Salud Pública de Barcelona, Spain; 2 Teaching Unit of Preventive Medicine and Public Health, PSMAR-UPF-ASPB, Barcelona, Spain; 3 CIBER en Epidemiología y Salud Pública (CIBERESP), Madrid, Spain; 4 Biomedical Research Institute Sant Pau (IIB Sant Pau), Barcelona, Spain; 5 Department of Pediatrics, Gynecology and Preventive Medicine, Universidad Autónoma de Barcelona, Spain; 6 Unitat Suport Metodològic a l'Investigació Biomedica (USMIB- Vall d'Hebron Institut de Recerca (VHIR), Universidad Autónoma de Barcelona, Spain

**Keywords:** Mandatory reporting, Disease notification, Population surveillance, HIV

## Abstract

**Background:**

In Catalonia, a law was passed in 2010 to incorporate HIV infection as a mandatory disease and to reduce under-reporting, perform follow-up and to improve prevention. Currently, there are studies that describe the surveillance of new diagnoses of HIV infection. However, there are no studies that compare the change from voluntary to mandatory notification. This study evaluates the impact of mandatory notification on the registered cases of newly diagnosed HIV infections in a city with a concentrated epidemic.

**Methods:**

We analysed newly diagnosed HIV infections that were included in the city register. A descriptive analysis compared the number and the epidemiological characteristics of cases that were declared in two different periods (when notification was voluntary in 2001–2009 and when mandatory in 2010–2011). Time series analysis was conducted, evaluating trends and changes by fitting a Poisson regression model. The Epidemiology Service from the Public Health Agency was responsible for gathering and analyzing data and producing reports on communicable disease for the city. The data used in this study is openly available.

**Results:**

Overall, 4510 cases of HIV infection were registered, 81.9% were men and 74.5% of them aged over 30. Among men, 55.6% were men who had sex with men (MSM), and among women, the most common route of transmission was heterosexual (HTS) with 65.4%. An annual average of 560 cases was registered between 2010 and 2011. This represents an increase of 33% from the annual average over the previous period (p<0.001). Time series analysis showed that the probability of notification was 2.8 (95% confidence interval 2.4-3.3) times higher with mandatory notification than in the earlier period. There was a statistically significant decrease of missing values in the period of mandatory notification (p<0.001).

**Conclusions:**

Mandatory notification of HIV has resulted in an increase in detection of newly diagnosed infections, reduced the levels of missing data and has provided a more realistic picture of the epidemiology of HIV. This information also helps to improve the suitability of interventions aimed at HIV prevention and control.

## Background

With the introduction of highly active antiretroviral treatments (HAART) in 1996, when HIV tests were largely available, the Acquired Immunodeficiency Syndrome (AIDS) ceased to be a good epidemiological marker for HIV infection [1]. A change occurred in the natural course of the infection, prolonging the period between infection and development of AIDS, as well as an important reduction in mortality [1]. As a consequence, data from AIDS registries no longer reflected the magnitude nor the characteristics of the epidemic [1,2]. Since 1999, the World Health Organisation (WHO) has promoted the implementation of population-based surveillance systems, for the new HIV cases in developed countries [3]. Since the notification is used in only some areas, this does not allow describing the general epidemiological characteristics of the infection [4].

Despite being one of the most affected Western European countries in terms of HIV infection rates, Spain does not have state-wide population-level data on HIV infection [2,5,6]. Epidemiological surveillance has been carried out voluntarily, based on the notification of new diagnoses of HIV infection by the various Autonomous Communities [7] and among them, Catalonia, which began notification of new cases in January 2001. It should be taken into account that levels of under-reporting could be around 15 to 30% [8]. Moreover, voluntary notification systems cannot ensure the elimination of duplicates and there is no follow-up until the development of AIDS or death. In 2010, Catalonia passed a law which incorporates HIV infection as an individually name-based mandatory disease [9] with the aim of reducing under-reporting, performing follow-up and improving prevention in the same way that AIDS had been since 1987 [10,11].

A total of 28 of the 30 countries belonging to the European Union were notifying new cases of HIV in 2009. In 2000, 13 of these countries had national notification systems and in 8 of them, notifications were mandatory [12,13]. Despite the existence of these reporting systems, there are no studies published evaluating the impact of the change from voluntary to mandatory notification.

The objective of this study was to evaluate the impact of mandatory notification on the numbers of new diagnoses of HIV infections reported in a large city with a concentrated epidemic; comparing the epidemiological characteristics of those cases registered before to those registered after the mandatory notification came into effect.

## Methods

### Study design and study population

The city of Barcelona is situated in the northeast of Spain. It had a population of 1,619,337 inhabitants in 2010 [14]. AIDS rates were 46.7 per 100,000 inhabitants in 1994, before the introduction of HAART, and 4.5 per 100,000 inhabitants in 2010 [10,11]. This city has a low-level of concentrated epidemic (where high levels of infection are found only in specific groups) and estimates that HIV prevalence is mainly based on data collected from populations most at risk – especially men who have sex with men (MSM) [3].

We analysed registered cases of new diagnoses of HIV infection in Barcelona over the period from 1 January 2001 to 31 December 2011, excluding AIDS diagnoses.

A structured questionnaire was used to collect clinical and epidemiological information: sex, age, region of birth, route of transmission, CD4 cell count and reporting centre. Doctors reported new diagnoses of HIV. The Epidemiology Service from the city’s Public Health Agency was responsible for gathering and analyzing data and producing reports on communicable disease for the city, therefore the data used in this study is openly available. Other changes, such as testing policy, campaigning to promote HIV testing or change of case definition, were not accompanied in the switch from voluntary to mandatory notification.

### Statistical methods

A descriptive analysis was carried out, comparing the number and epidemiological characteristics of cases registered voluntarily from 2001 to 2009, with those corresponding to the period of mandatory notification from 2010 to 2011, by means of the chi-squared test. A Poisson regression model was fitted to analyse the time series. Quarter was considered as a unit of time, taking the first period as reference. In order to test trend, a linear component was fitted, adding a quadratic function that allows for attenuation of trend over time. A dummy variable indicating the mandatory period was then included, in order to evaluate the magnitude of the effect of the law coming into force on the number of newly notified diagnoses. Finally, an interaction term was included to allow a change in the incidence trend in the second period. The analysis was conducted on the total number of cases, as well as stratified by demographic variables, transmission route and reporting centre. Furthermore, interactions between quarter and the stratifying variables were tested. The likelihood test was used to compare models, and only statistically significant results were included in the final models. 95% confidence intervals (CI) and p-values < 0.05 were considered statistically significant. All analyses were carried out with SPSS version 18 [15] and STATA version 11.0 [16].

## Results

During the study period, 4510 new diagnoses of HIV infection were notified, representing an average of 410 cases annually. The epidemiological characteristics of new diagnoses reported are showed in Table 1. 81.9% were men, and 74.5% were aged over 30 years. By sex, only 43% of the men and 32.5% of the women were born in Spain. Among men, 45.5% were MSM, 10.8% were heterosexuals (HTS), and 9.3% were intravenous drug users (IDU). Among women, the most common route of transmission was HTS (65.4%), followed by IDU (15.2%). At the time of their diagnosis, 39.3% had CD4>350 cell/ul, 14.7% between 350 and 200 cell/ul, 12.9% under 200 cell/ul and 33.3% unknown. The most common reporting centre was a hospital, accounting for 64.6% of all reported cases.

**Table 1:** Epidemiological characteristics of HIV new diagnoses reported in Barcelona (2001 – 2011)

		**2001-2011**		**2001-2009 Voluntary period**		**2010-2011 Mandatory period**		P-value*
		**n=(4510)**		**n=(3390)**		**n=(1120)**		
		**N**	**%**	**N**	**%**	**N**	**%**	
**Sex**	Men	3692	81,9	2713	80.0	979	87.4	<0.001
	Women	818	18,1	677	20	141	12.6	
**Age (<30 and >30 years age)**	<30 years of age	619	13,7	289	8,5	330	29.5	<0.001
	>30 years of age	3358	74,5	2574	76	784	70	
	Unknown	533	11,8	527	15.5	6	0.5	
**Age groups**	14-19 years of age	9	0,2	0	0	9	0.8	<0.001
	20 -29 years of age	474	10,5	207	6.1	267	23.8	
	30-39 years of age	1562	34,6	1117	32.9	445	39.7	
	40-49 years of age	1306	29,0	1021	30.1	285	25.4	
	50-59 years of age	418	9,3	343	10.1	75	6.7	
	> 60 years of age	208	4,6	175	5.2	33	2.9	
	Unknown	533	11,8	527	15.5	6	0.5	
**Region of birth**	Spain	1854	41,1	1304	38.5	550	49.1	<0.001
	Western Europe and North America	281	6,2	174	5.1	107	9.6	
	Latin America and Caribbean	849	18,8	561	16.5	288	25.7	
	Eastern Europe and Central Asia	145	3,2	86	2.5	59	5.3	
	North Africa and Middle East	54	1,2	37	1.1	17	1.5	
	Sub-Saharan Africa	198	4,4	148	4.4	50	4.5	
	Unknown	1129	25,0	1080	31.9	49	4.4	
**Route of transmission**	HTS men	487	10,8	359	10.59	128	11.43	<0.001
	HTS women	536	11,9	429	12.65	107	9.55	
	MSM	2052	45,5	1387	40.91	665	59.38	
	IDU men	419	9,3	334	9.85	85	7.59	
	IDU women	124	2,7	103	3.04	21	1.88	
	Unknown	892	19,8	778	23,0	114	10.18	
**CD4 count**	> 350 cell/ul	1773	39,3	1257	37.1	516	46.1	<0.001
	< 350–200 cells/ul	665	14,7	469	13.8	196	17.5	
	<200 cells/ul	580	12,9	436	12.9	144	12.9	
	Unknown	1492	33,1	1228	36.2	264	23.6	
**Reporting centre**	Hospital	2915	64,6	2060	60.8	855	76.3	<0.001
	Primary care	1595	35,4	1330	39.2	265	23.7	

Comparison between voluntary notification period with mandatory notification period. (2001–2009 versus 2010–2011).

HTS: Heterosexual contact, MSM: Men having sex with men, IDU: Injecting drug Users.

* Chi^2^ test.

The annual average of notifications over the period 2001–2009 was 376.6, while that for 2010–2011 was 560, representing a 33% increase in the annual number of notifications (p <0.001). The epidemiological characteristics of the cases by period are showed in Table 1. It was observed that there was a change in the percentage composition by sex, with an increase in the proportion of notifications corresponding to men. The majority of cases were aged over 30 years. However, a change was seen in the proportion of notifications among individuals aged under 30, which rose from 8.1% to 29.5% (p<0.001). With regard to region of birth, those born in Spain presented a decline in notifications, which led to an increase in the relative proportions of foreign-born individuals, the most common of which were people born in Latin American and Caribbean countries. The rate of notification corresponding to cases of sexual transmission rose from 64.2% to 80.4% (p<0.001), particularly among MSM whereas among IDU, there was a decline from 12.9 to 9.5%. There was a significant decline in the proportion of missing values for most of the variables during the period of mandatory notification, especially region of birth and route of transmission, comparing with the voluntary period (p<0.001).

**Figure 1:**
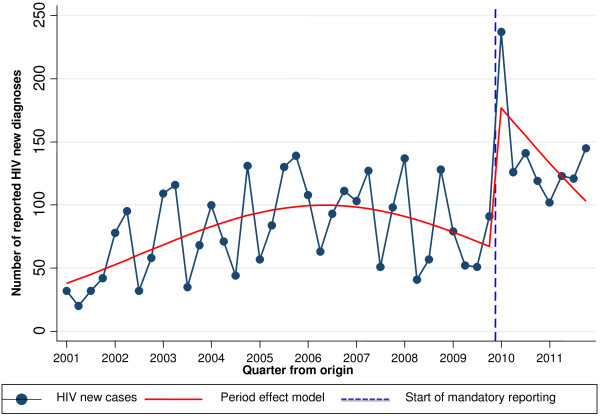
Evolution of new HIV diagnoses notified in Barcelona, between January 2001 and December 2011.

As shown in Table 2, the probability of notification increased by 2.8 times (CI 2.4-3.3) with the introduction of mandatory HIV notification, compared to the period when notification was voluntary. Figure 1 shows a rise in the number of cases of HIV notified up to the year 2006, a year in which a change in the trend may be seen, after which cases began to decline. With the introduction of mandatory notification, an increase is observed in the level of cases notified. However, the decreasing trend of series of quarters is not altered by the change in notification, since the interaction between the trend and the law coming into force was not significant.

**Table 2:** Effect of the introduction of mandatory notification of new HIV diagnoses in Barcelona

	**RR**	**CI 95%**	**P-value**
**Linear Trend**	1.09	1.08-1.10	<0.001*
**Quadracticadjustmentterm**	0.99	0.99-0.99	<0.001*
**Indicator of introduction of mandatory notification**	2.79	2.39-3.25	<0.001*

Poisson Regression N cases= α+β_1_(Quarter from origen) + β_2_(Quarter from origen)^2^ + β_3_(mandatory notification indicator).

RR: relative risk, CI: confidence interval.

^*^ Poisson Regression.

**Figure 2:**
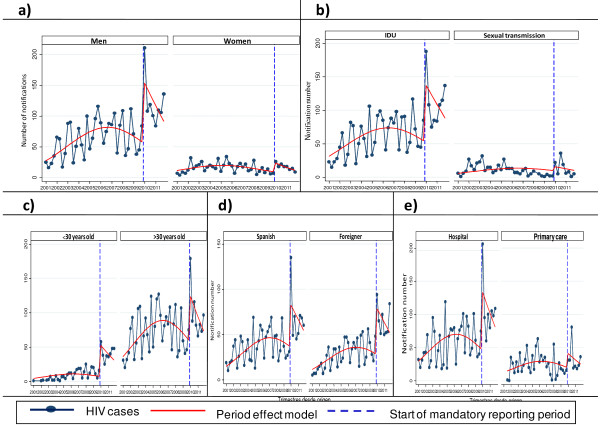
**Reporting of new HIV diagnoses by quarter.** Stratified by sex (**a**), route of transmission (**b**), age (**c**), region of birth (**d**), and reporting center (**e**). Barcelona, January 2001 – December 2011.

Figure 2 illustrates the increase in HIV notified cases, following the introduction of the law, in terms of sex, age, region of birth, route of transmission and reporting centre. Only the interaction between sex and quarter was statistically significant (p <0.001), suggesting that the trend differs between men and women and that, in men, the decrease in HIV reporting was greater than in women in the period of voluntary notification.

## Discussion

In Barcelona, AIDS has been a mandatory notified disease since 1986, and HIV was included in the registry as a voluntary notifiable disease only until 1996 [8]. During the HIV/AIDS epidemic there was, and continues to be, controversy over whether HIV infection should be notified. Following a long debate over many years, in 2010 Catalonia passed a law making a mandatory registry of HIV infection [8]. Such a mandatory registry permits better characterisation of the infection, avoidance of duplication, the study of sexual partners, effectiveness of therapy, and follow-up of cases of infection until the appearance of AIDS, or death [17]. However, some studies have suggested that the introduction of this type of notification could diminish the number of persons who undergo the test; however these predictions have not been reflected in significant declines in testing in the United States [18].

In our study, both the results of bivariate analysis and of time series analysis showed that the introduction of mandatory notification of new diagnosis of reported HIV infection led to an increase in such notification of almost three times. This increase in the completeness of notification results in a more precise description of the epidemiological situation regarding HIV infection. The epidemiological characteristics are similar in both periods, although some proportions have changed, new diagnoses being more common among men with higher risk of sexual transmission; particularly MSM and those aged over 30 years. There is a notable percentage increase of groups at risk following the introduction of the law, such as individuals aged under 30 and people born outside Spain. Regarding the notifying centre, hospitals are the main sources of notifications in both periods.

Comparison of the epidemiological characteristics against data available at national level (2003–2010) revealed differences with respect to the proportions in the different categories of transmission. It is important to stress that national-level data cover 71% of the population (32,843,416 inhabitants in 2010) [19], making it difficult to extrapolate to the rest of the country, since the degree and patterns of HIV transmission differ between the different Autonomous Communities. It should also be noted that amongst them, the notification of new cases of HIV is voluntary and that some proportion of these cases have probably not been notified [19]. This reveals the need to have a notification system that permits more exhaustive surveillance of the epidemic, in order to obtain appropriate quality and quantity of information. Thus, this information will allow the estimation of the magnitude of the infection and to monitor its trends [20], especially in the large cities, which is where cases tend to be concentrated. Comparing with the data of HIV diagnoses that were reported by the EU/EEA countries in 2011 (rate of 5.7 per 100000 in the population), 10% of all HIV diagnoses were reported among young people aged 15 to 24 years, and 30% among MSM [21]. These trends are similar to those observed in Barcelona, describing a concentrated epidemic especially by sexually transmitted infection, mainly in MSM.

Studies with regard to the switch from voluntary to mandatory notification were not possible to find. However, in other settings, it has been observed that appropriate and exhaustive systems of surveillance of transmissible infections, not only HIV, generate more precise information at population level, presenting a more realistic picture of the infection situation [22,23]. Accurate information has helped large scale efforts in eradicating smallpox [24] and reducing dracunculiasis in Asia and Africa [25,26].

Reporting of HIV infection must be interpreted with caution however, taking into account other available epidemiological data, because these reports do not provide a direct measurement of the incidence or prevalence of HIV infection [12,13]. The proportion of HIV infected individuals, who are diagnosed and reported, varies according to the phase of the epidemic [27], HIV testing patterns [28], and characteristics of surveillance systems. Therefore, new diagnoses of HIV infection, reported into a registry, varies despite of their mandatory or voluntary state. But with a mandatory notification registry, all cases that have been diagnosed are reported, despite their infection state. HIV mandatory reporting is helping to improve assessment of the scale and extent of recent HIV transmission in the population [12], to visualize a better picture of HIV endemic situation in the city and to describe the epidemiological characteristics of emerging risk groups. It is important to stress that with mandatory notification, elimination of duplicate reports and the ability to match reports of HIV infection with other data sets are accomplished [12]. These are two characteristics [3], which are essential requirements, for an effective HIV reporting system. Another important advantage of mandatory notification is that it is possible to recover data by active surveillance; therefore there might be a decrease in missing data as it was observed in our analysis.

### Strengths and limitations

Time series analysis has permitted the description and estimation of the evolution in trends affecting the notification of new diagnoses of HIV infection [29,30]. Thus, while on the one hand we observe an increase in the number of mandatory notifications, the slope of the overall trend persists in both periods.

Among the limitations of this study, one is the short period of follow-up of mandatory notification. This raises the issue of the need to continue its evaluation, in order to determine whether the changes and the rise in number of notifications persist over time.

Another aspect to be taken into account is that the analysis was conducted with the start date of mandatory notification as January 2010, despite the fact that the law only came into force in July 2010, and the publication of the 2010 manual of notifiable diseases and mandatory notification of HIV infection was also included [31]. Consequently, some centres, not notified when this was voluntary, began to do so at the beginning of 2010. We decided that the first two quarters of 2010 were included in the analysis, as the impact of mandatory notification starts from the second quarter of this year, despite the law only coming into force in July 2010.

## Conclusions

In conclusion, while the voluntary system of notification was useful in order to describe global trends of the infection, HIV mandatory notification increased the number of new diagnoses notified, made visible a change in the relative proportions of epidemiological characteristics of the HIV infection, such as greater representation of men (particularly the MSM group), has led to the appearance of certain groups that were formerly not so strongly represented, such as individuals aged under 30 years. This information, more exhaustive and of a higher quality than that obtained with the voluntary notification system, will serve to improve evaluation and design of prevention programmes allowing more effective control of HIV infection.

## Abbreviations

AIDS: Acquired Immunodeficiency Syndrome; CI: confidence intervals; HAART: highly active antiretroviral treatments; HIV: Human immunodeficiency virus; HTS: heterosexuals; IDU: intravenous drug users; MSM: men who have sex with men; RR: relative risk; WHO: World Health Organisation.

## Competing interests

The authors declare that they have no competing interests.

## Authors' contributions

JMR, as the main author, made substantial contributions to the conception, design and interpretation of the data. PGO, SPH and JAC have all been involved in the designing of the study, drafting the manuscript, or revising it critically for important intellectual content. All authors read and approved the final manuscript.

## Pre-publication history

The pre-publication history for this paper can be accessed here:

http://www.biomedcentral.com/1471-2458/13/338/prepub

## References

[B1] Hamers FF Infuso A Alix J Downs AM Current situation and regional perspective on HIV/AIDS surveillance in Europe J Acquir Immune Defic Syndr 2003 32 Suppl 1 S39 48 12571514 10.1097/00126334-200302011-00007

[B2] Kaldor JM Delpech V Guy RJ AIDS case reporting: do we still need it? Lancet 2009 373 181 183 10.1016/S0140-6736(08)61006-5 18684500

[B3] UNAIDS/WHO: Guidelines for second generation HIV surveillance [http://www.who.int/hiv/pub/surveillance/en/cds_edc_2000_5.pdf] 24049865

[B4] Devaux I Alix J Likatavicius G Herida M Nielsen S Hamers FF Nardone A Human immunodeficiency virus (HIV) and acquired immunodeficiency syndrome (AIDS) case reporting in the World Health Organization European Region in 2006 Euro Surveill 2008 13 39 pii=18988 18822241

[B5] Hamers FF Phillips AN Diagnosed and undiagnosed HIV-infected populations in Europe HIV Med 2008 9 Suppl 2 6 12 18557863 10.1111/j.1468-1293.2008.00584.x

[B6] Peersman G Rugg D Erkkola T Kiwango E Yang J Are the investments in national HIV monitoring and evaluation systems paying off? J Acquir Immune Defic Syndr 2009 52 Suppl 2 S87 96 19901631 10.1097/QAI.0b013e3181baede7

[B7] Ministerio de Sanidad, Servicios Sociales e Igualdad - Ciudadanos - Vigilancia epidemiológica. [Ministry of Health, Social Services and Equality - Citizens - Surveillance] [http://www.msps.es/ciudadanos/enfLesiones/enfTransmisibles/sida/vigilancia/home.htm] [in spanish]

[B8] Casabona J Romaguera A Almeda J Blanch C Caylà JA Miró JM Colom J Reporting new HIV cases in Catalonia, Spain: is technical consensus feasible? Gac Sanit 2003 17 1 75 82 12605750 10.1016/s0213-9111(03)71695-1

[B9] Boletín Oficial del Estado Decreto 67/2010, de 25 de mayo, por el que se regula el sistema de notificación de enfermedades de declaración obligatoria y brotes epidémicos en el Departamento de Salud. [Decree 67/2010, 25th May, regulating the notification system of notifiable diseases and outbreaks in the Department of Health] DOGC. Diario Oficial de la Generalitat de Catalunya [in spanish] 2010 5666 8 Jul

[B10] Agencia de Salud Pública de Barcelona, Epidemiology Service Sida en Barcelona, Vigilancia epidemiológica, reporte 89 [AIDS in Barcelona, Epidemiological surveillance, report 89] 2011 89 Barcelona, Spain: Agencia de Salud Pública de Barcelona 1 20 [http://www.aspb.es/quefem/docs/sida89.pdf]. [in spanish]

[B11] Generalitat de Catalunya, Departament de Salut: Sistema de declaración de VIH e ITS en Cataluña 2010. [Declaration system of HIV and STI in Catalunya 2010] Generalitat de Catalunya, Departament de Salut: Sistema de declaración de VIH e ITS en Cataluña 2010. [Declaration system of HIV and STI in Catalunya 2010] [http://www.sidastudi.org/resources/inmagic-img/DD9815.pdf] [in catalan]

[B12] Infuso A Hamers FF Downs AM Alix J HIV reporting in western Europe: national systems and first European data Euro Surveill 2000 5 2 pii=29 10.2807/esm.05.02.00029-en 12631871

[B13] Van de Laar MJ Likatavicius G Stengaard AR Donoghoe MC HIV/AIDS surveillance in Europe: update 2007 Euro Surveill 2008 13 50 pii=19066 10.2807/ese.13.50.19066-en 19087870

[B14] Instituto Nacional de Estadística. Revisión del padrón municipal 2011 [Review of 2011 municipal census] [http://www.ine.es/jaxi/tabla.do] [in spanish]

[B15] SPSS for Windows; Version 18 2009 Chicago, Illinois: SPSS Inc.

[B16] Stata Statistical Software Stata Corp 2011 College Station, TX: Stata Corp LP Release 12

[B17] Hamers FF Recommendations for HIV surveillance in Europe Euro Surveill 1998 3 5 pii=116 10.2807/esm.03.05.00116-en 12631771

[B18] Nakashima AK Horsley R Frey RL Sweeney PA Weber JT Fleming PL Effect of HIV reporting by name on use of HIV testing in publicly funded counseling and testing programs JAMA 1998 280 16 1421 1426 10.1001/jama.280.16.1421 9801002

[B19] Área de Vigilancia de VIH y Conductas de Riesgo. Vigilancia Epidemiológica del VIH/sida en España Sistema de Información sobre Nuevos Diagnósticos de VIH y Registro Nacional de Casos de Sida. Secretaría del Plan Nacional sobre el Sida/Centro Nacional de Epidemiología. [Information System on new HIV diagnoses and National Register of AIDS cases. Secretariat of the National AIDS Plan / National Epidemiology Center] [http://www.msc.es/novedades/docs/InformeVIH-sida_Junio2011.pdf] [in spanish]

[B20] Pisani E Lazzari S Walker N Schwartlander B HIV surveillance: a global perspective J Acquir Immune Defic Syndr 2003 32 Suppl 1 S3 11 12571509 10.1097/00126334-200302011-00002

[B21] Likatavicius G MJ Vdl HIV infection and AIDS in the European Economic Area 2010 Euro Surveill 2011 16 48 pii=20030 22172299

[B22] Diaz T De Cock K Brown T Ghys PD Boerma JT New strategies for HIV surveillance in resource-constrained settings: an overview AIDS 2005 19 Suppl 2 S1 8 15930836 10.1097/01.aids.0000172871.80723.3e

[B23] Boerma JT Weir SS Integrating demographic and epidemiological approaches to research on HIV/AIDS: the proximate-determinants framework J Infect Dis 2005 191 Suppl 1 S61 7 15627232 10.1086/425282

[B24] Lane JM Mass vaccination and surveillance/containment in the eradication of smallpox Curr Top Microbiol Immunol 2006 304 17 29 10.1007/3-540-36583-4_2 16989262 PMC7120753

[B25] Hopkins DR Ruiz-Tiben E Downs P Withers PC Maguire JH Dracunculiasis eradication: the final inch Am J Trop Med Hyg 2005 73 4 669 675 16222007

[B26] Richards F Hopkins D Surveillance: The foundation for control and elimination of dracunculiasis in Africa Int J Epidemiol 1989 18 4 934 943 10.1093/ije/18.4.934 2533584

[B27] Smith E Jensen L Wachmann CH Patterns and trends in clinically recognized HIV seroconversions among all newly diagnosed HIV-infected homo-/bisexual men in Denmark, 1991–1994 AIDS 1996 10 765 70 8805868 10.1097/00002030-199606001-00011

[B28] De Cock KM Johnson AM From exceptionalism to normalisation: a reappraisal of attitudes and practice around HIV testing BMJ 1998 316 292 5 10.1136/bmj.316.7127.290 PMC2665487 9472517

[B29] Saez M Perez-Hoyos S Tobias A Saurina C Barcelo MA Ballester F Time series methods in epidemiological studies on air pollution Rev Esp Salud Publica 1999 73 2 133 143 10.1590/S1135-57271999000200004 10410597

[B30] Iniguez C Ballester F Perez-Hoyos S Saez M Estimate of daily cases of influenza from the cases notified to the Reportable Disease System: usefulness in time series studies Gac Sanit 2001 15 3 273 275 10.1016/S0213-9111(01)71559-2 11423034

[B31] Generalitat de Catalunya Definició de cas de les Malalties de declaració obligatória [Case definition of notifiable diseases] 2011 5 Barcelona: Generalitat de Catalunya, Departament de salut [in catalan]

